# Pharmacological Aspects of Over-the-Counter Opioid Drugs Misuse

**DOI:** 10.3390/molecules25173905

**Published:** 2020-08-27

**Authors:** Łukasz Sobczak, Krzysztof Goryński

**Affiliations:** Bioanalysis Scientific Group, Faculty of Pharmacy, Collegium Medicum in Bydgoszcz at Nicolaus Copernicus University in Toruń, 87-100 Toruń, Poland; lukasz.sobczak@cm.umk.pl

**Keywords:** over-the-counter drugs, misuse, abuse, opioid drugs, pharmacology, codeine, dihydrocodeine, loperamide

## Abstract

Several over-the-counter (OTC) drugs are known to be misused. Among them are opioids such as codeine, dihydrocodeine, and loperamide. This work elucidates their pharmacology, interactions, safety profiles, and how pharmacology is being manipulated to misuse these common medications, with the aim to expand on the subject outlined by the authors focusing on abuse prevention and prevalence rates. The reviewed literature was identified in several online databases through searches conducted with phrases created by combining the international non-proprietary names of the drugs with terms related to drug misuse. The results show that OTC opioids are misused as an alternative for illicit narcotics, or prescription-only opioids. The potency of codeine and loperamide is strongly dependent on the individual enzymatic activity of CYP2D6 and CYP3A4, as well as P-glycoprotein function. Codeine can also be utilized as a substrate for clandestine syntheses of more potent drugs of abuse, namely desomorphine (“Krokodil”), and morphine. The dangerous methods used to prepare these substances can result in poisoning from toxic chemicals and impurities originating from the synthesis procedure. OTC opioids are generally safe when consumed in accordance with medical guidelines. However, the intake of supratherapeutic amounts of these substances may reveal surprising traits of common medications.

## 1. Introduction

Over-the-counter (OTC) drugs are medicines sold without medical prescription to treat common and temperate medical conditions. Unfortunately, the misconception that OTC drugs are devoid of any harm to users has become established as a commonly held belief. While it is true that most of them are relatively safe, if administered with moderation, misuse is usually associated with the intake of excessive amounts and is burdened with life-threatening consequences. Due to the acknowledged misuse liability, or associated health risks, some countries have already restricted access to several OTC drugs by introducing an intermediate category of pharmacy-only (or pharmacist-only) medicines (POMs). While the purchase of POMs does not require a prescription from a physician, they may only be purchased in a pharmacy. Other restrictions, such as age limit or maximal purchase quotas, may also be in place for the sale of POMs and OTC drugs.

This matter is further complicated by the differences in local regulations. For example, codeine is available as an OTC medicine in countries such as Denmark [1], Poland (up to 240 mg per single purchase—since December 2016) [2], the UK (up to 12.8 mg per single tablet), and several other European states [3], as well as in Japan. At the same time, it is classified as prescription only medicine in Australia (where it has been recently up-scheduled from the OTC category) [4], or USA [5]. Dihydrocodeine, a stronger opioid drug, is generally not available as an OTC medicine. However, few exceptions exist—e.g., in the UK or Japan. Loperamide on the other hand, is usually available without prescription and without almost any restrictions regarding its sale.

All of the aforementioned drugs are classified as opioid agents, and the evidence exists that they are misused, either unintentionally, or for non-medical intents. Several authors have already investigated the issue of misuse and abuse of OTC drugs from the perspective of pharmacology; however, these reports usually address single drugs, and few reports that are focused on a broader picture are regretfully still not fully comprehensive. This especially concerns the opioid drugs that tend to be omitted from such reviews. Out of four of the most extensive works investigated by the authors, only two discuss codeine, and none discuss either dihydrocodeine or loperamide [6,7,8,9].

This review is focused on three opioid drugs—codeine, dihydrocodeine, and loperamide—that can still be purchased without medical prescription in numerous parts of the world, addressing their pharmacology, interactions, safety profiles, and how pharmacology is manipulated in non-medical applications. This work intends to elucidate the reasons behind the misuse or abuse of these common medications. As such, it adds to numerous works regarding abuse prevention and prevalence rates that are already published.

## 2. Results and Discussion

### 2.1. Introduction to Opioid Drugs

From a chemical standpoint, opioids comprise a diverse group of drugs, but they all share a common affinity towards µ, δ, and κ opioid receptors. Most of the opioids used in clinical practice, including those available as OTC medicines, are agonists of opioid receptors that are predominantly selective for µ type receptors. Receptor type specific effects, as well as some examples of the drugs that are selective ligands for those receptors, are presented in Table 1 [10,11].

Therapeutically beneficial analgesia results from diminished nociceptor excitability and the reduced release of pro-inflammatory peptides at nerve terminals [12]. However, effects, such as euphoria resulting from the agonism of µ receptors (described as sudden rush), mood modulation contributed by the agonism of δ receptors, or hallucinations caused by agonism of κ receptors [10,11], are often credited with the interest in these drugs with non-medical intents. Opioids also possess synergic effects with GABAergic receptor agonists, such as alcohol, barbiturates, or benzodiazepines.

Typical opioid overdose is associated with a characteristic triad of symptoms: decreased consciousness (or coma), abnormally slow or ceased respiration, and pinpoint pupils. Respiratory depression can manifest as cyanosis and have severe (neural damage caused by cerebral hypoxia) or even fatal consequences [10]. Paraesthesia (abnormal dermal sensations), urinary retention, and histamine-mediated reactions (emesis, flushing, itching, and nausea) have been also reported for opiate use [23]. Prolonged abuse results in physical and psychological dependence, the development of tolerance (possibly due to the desensitization and internalisation of µ receptors [24], and probable formation of opiate antibodies [25]), and is associated with the onset of withdrawal syndrome (symptoms include: agitation, diarrhoea, insomnia, muscle cramps, panic attacks, and sweating), when dosing is abruptly discontinued [26,27].

### 2.2. Codeine

Codeine is a natural alkaloid of opium poppy (an opiate), with affinity to µ, δ and κ receptors acting as their agonist. Codeine is approximately 20 times more selective towards the µ receptors than towards δ type, and even less selective (ca. 20–30 times) towards κ receptors (see Table 1). It is marketed as a cough suppressant and analgesic and is available for patients as tablets and syrups. In medications indicated for mild to moderate pain control, codeine is often paired with paracetamol (acetaminophen) or ibuprofen, and in medications indicated for cough and cold with promethazine or salicylic acid. The drug is generally not perceived as harmful by the patients [28,29], and this lenient attitude has perhaps resulted in increased worldwide consumption within the last 20 years and also in increased codeine dependency rates. In this regard, codeine is the world’s most consumed opioid (based on drug quantity [30]), and codeine-dependent individuals account for approximately 2% of all admissions to some substance abuse centers [31].

**Figure 1:**
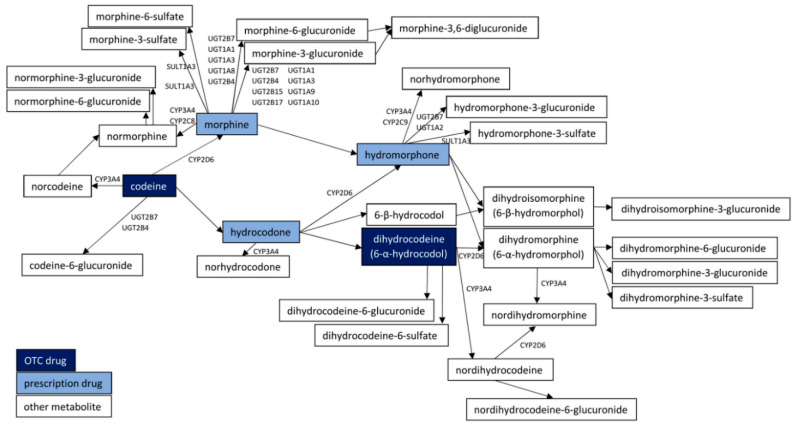
Metabolic pathways of codeine and dihydrocodeine. Abbreviations used: CYP = cytochrome P450, SULT = sulfotransferase, UGT = uridine diphosphate glucuronosyltransferase.

The pharmacological profile of codeine as therapeutic drug, as well as a misused substance, is an outcome of active metabolite formation. The complex biotransformation pathways leading to prescription-only opioids, such as hydrocodone (up to 11% of parent drug), morphine (5–30%), and its more potent metabolites (hydromorphone and morphine-6-glucuronide), are presented in Figure 1 [6,12,32]. Such pharmacokinetics result in codeine potency being approximately one tenth the potency of morphine and resulting equianalgesic dose of codeine being 6.67–10 times larger than with morphine (both administered orally) [33,34].

Although codeine is almost completely absorbed from the gastrointestinal tract (94 ± 4%), the pharmacokinetics of the drug may vary significantly between individuals due to genetic polymorphisms of *CYP2D6* and *CYP3A4* genes [6]. The transformation of codeine to morphine is especially dependent on CYP2D6 functionality, a factor firmly associated with one’s ethnicity. In poor metabolizers (with two non-functional alleles of the *CYP2D6* gene), codeine may not express the desired effect at all, while in ultra-rapid metabolizers (with three or more functional alleles), poisoning is possible even within the recommended dose [12,32,35,36]. Other factors, such as a fat-rich diet and contents of grapefruit juice (especially bergamottin, an inhibitor of CYP3A4), are proven to potentialize the drug. In addition, P-glycoprotein (P-gp)—an efflux pump preventing certain drugs from penetrating the blood–brain barrier (BBB)—has been shown to have significant impact on brain concentration of the morphine [37].

As a cough suppressant, codeine (through active metabolites) acts in sub-analgesic doses at the µ_2_ and κ receptors by inhibiting the medullary cough center. The analgesic action is mediated by µ receptors, with the complementary contribution of other opioid receptor types [6,38]. Codeine-containing OTC medications are often misused to achieve euphoria, relaxation, and feeling of warmth (described as recurrent waves of pleasurable sensations), or as a substitute for illicit narcotics [26]. In a single dose, codeine is generally described by its misusers as sedating, but repetitive doses tend to be energizing [27]. Positive reinforcement is an effect of µ_2_ receptor stimulation. Misuse has also been reported as a means to escape persistent pain or achieve a state of disconnection or dissociation. This dissociative state is described as a dream-like floating sensation accompanied by slight hallucinations (e.g., seeing geometric shapes), or even out-of-body experiences [26].

The symptoms of codeine overdose are mostly consistent with typical opioid poisoning, but the drug originating from OTC formulations is often accompanied by non-opioid analgesics. Thus, hepatotoxicity from paracetamol, or gastrointestinal damage (haemorrhages, ulcerations, hypokalaemia, metabolic acidosis) and nephrotoxicity caused by ibuprofen are common consequences of its misuse [27,39,40,41]. In effort to evade these harms, various homemade extraction methods are used to separate codeine from the abovementioned drugs—for example, the so-called cold water extraction that exploits solubility differences between ingredients [35,42,43]. Lately, some pharmaceutical companies begun to incorporate physical or chemical barriers to their products as a means to combat such practices [26].

OTC codeine tablets are available in doses up to 15 mg, with guidelines recommending 15 mg every 4–6 h (maximally 45 mg a day) as an antitussive, and up to 15–30 mg every 6h (maximally 90 mg a day) for pain control. Drug misusers propose 30–60 mg as a starting dose (to test individual reaction for the drug), and further doses of 100–250 mg in order to achieve euphoria [27,28,44]. While a quantity of 500–1000 mg is usually considered lethal [45], as much as 1536 mg a day has been reported as dependent individuals’ routines [6,26,39,40,46,47,48]. Due to the large doses consumed, codeine used for non-medical purposes is more dangerous than morphine used for anesthesia. The safety ratio of misused codeine is ca. 20 [45] (ratio of usual lethal dose to the usual effective dose), while the therapeutic index of morphine is 70 [49] (ratio of lethal dose (LD_50_) to the therapeutic effective dose (ED_50_)). The safety ratio of codeine is equal to that of methadone (when both drugs are administered orally for non-medical purposes), but this is more than three times larger than for the injected heroin [45]. Based on animal studies it is possible to compare the safety of codeine with morphine, and also with fellow OTC opioid loperamide (all drugs administered orally; see Table 2). Experimental data show that, despite its reputation as a safe drug [28,29], codeine is in fact dangerous and is characterized by a narrow therapeutic index.

The effects of opioids such as codeine are known to be potentialized by the non-selective H_1_ histaminergic receptor antagonists such as promethazine. Usually, therapeutic doses of first-generation antihistamines cause sedation by blocking the H_1_ receptors located in the central nervous system (CNS), sometimes to such extent that diphenhydramine is even marketed as an OTC sleeping aid. Surprisingly, supratherapeutic doses may cause paradoxical CNS stimulation (euphory, hallucinations), mainly due to anticholinergic poisoning [59]. Promethazine misuse is either related to its calming effect or ability to induce paradoxical euphoria and occasional hallucinations (auditory and visual), when administered in larger amounts [60]. Such effects are further enhanced by the simultaneous consumption of alcohol. To exploit these features, promethazine–codeine containing syrup is mixed with alcohol, candy, and soft drinks to create “purple drank”. The concoction is probably named after the color of the syrup’s dye and pursued in order to achieve euphoric and relaxing sensations [6,61]. Arrhythmia (manifesting as a prolongation of the QT interval in electrocardiogram), delirium, psychoses, substance dependence, and withdrawal syndrome are some of the known consequences of its misuse, as well as tardive dyskinesia resulting from the antagonism of D_2_ dopaminergic receptors [62].

**Figure 2:**
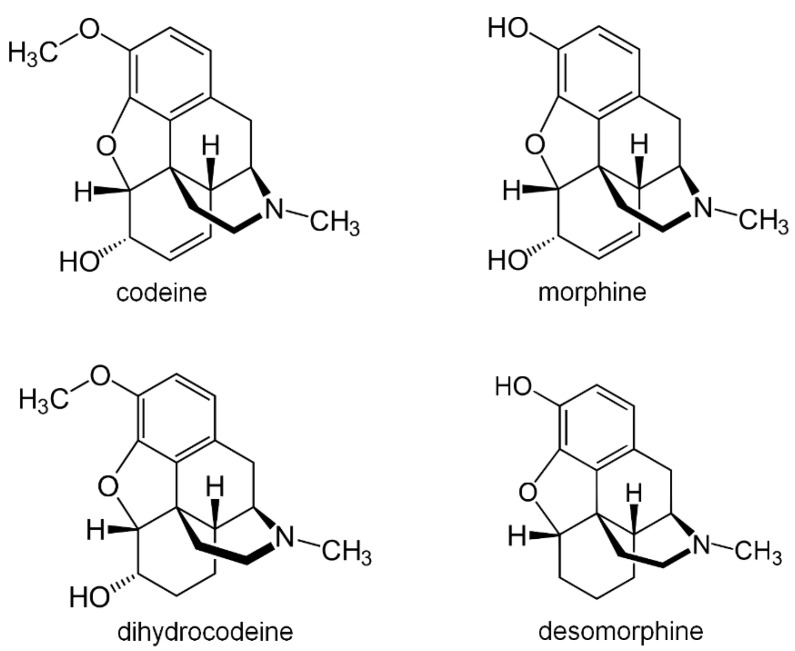
Chemical structures of selected opioid drugs.

Another alarming trend entails clandestine syntheses of more potent opioids with codeine used as a substrate. The “homebake” method results in the demethylation of codeine to morphine, while with a slightly more complex protocol [63,64,65,66] it is possible to obtain desomorphine—a drug with an 8–10 times stronger analgesic effect (up to 80–100 times stronger than the precursor codeine) and three times the toxicity of morphine [42,65]. Structural differences between these opioids are presented in Figure 2.

Desomorphine is commonly known as “Krokodil”, allegedly due to consequences of its injectable use, which presents as discoloured (green/black), flaking, and scale-like skin with ulcerations, somewhat resembling crocodile scale [11,23,63]. Although the drug is dated back to 1920s, it remained on the sidelines until its non-medical use spread across Russia, Ukraine, and neighbouring countries as a shocking new menace, reaching its apex just several years ago. Harm caused by “Krokodil” seems to be mostly related to reagents and contaminants introduced during the synthesis process, and include jaw osteonecrosis caused by red phosphorus, heavy metal poisoning (concentration, memory, motor, and speech impairments) due to corroded laboratory equipment, and skin infections with vascular damage that often leads to necrosis/gangrene, sometimes requiring limb amputation [23]. As an opioid, desomorphine has the same µ receptor affinity as morphine (but weaker to δ and κ type receptors), but is more addictive due a to faster onset of action and shorter elimination time, while also not inducing the emetic effects related to the use of morphine [64,67]. Damage to liver and kidneys has also been reported, as well as hallucinations [23].

### 2.3. Dihydrocodeine

Twice as strong as codeine, dihydrocodeine is a cough suppressant and analgesic used for mild to moderate pain control [68]. Slight euphoria, reported as a therapeutic side effect, increases further with supratherapeutic doses. Other adverse effects include constipation, drowsiness, dry mouth, headaches, nausea, respiratory depression, urinary retention, and substance dependence.

Dihydrocodeine, an agonist of µ, δ, and κ opioid receptors, is highly selective for µ type (µ/δ selectivity ratio is ca. 20–25; µ/κ selectivity ratio is ca. 45–55; see Table 1). As with codeine, dihydrocodeine acts through the formation of active metabolites. A small percentage of parent drug (1.3–9%) is transformed to dihydromorphine (1.2 times as potent as morphine) by CYP2D6. However, the formation of this metabolite is not solely responsible for the analgesic effect of dihydrocodeine [69]. Other metabolites include nordihydrocodeine, which has the same µ, δ, and κ receptors affinity as dihydrocodeine (16% of parent drug, mediated by CYP3A4), and dihydrocodeine-6-glucuronide (28% of parent drug) [68,70,71].

Over-the-counter dihydrocodeine medicines are available as syrups and tablets with doses up to 20 mg, and often contain paracetamol, or non-steroidal anti-inflammatory drugs (NSAIDs), as opposed to prescription-only medications, in which dihydrocodeine is generally the sole active ingredient, which is also present in higher dose and in controlled release formulation. Typical dihydrocodeine dosing as an OTC analgesic is ca. 7.5–15 mg every 4–6 h, not surpassing ca. 60 mg a day. Reports on drug misuse point at single doses of 300–900 mg, and up to 1350 mg a day [72]. A single dose of 70 mg is usually sufficient to induce a euphoric state. The lowest published toxic dose (oral) is 28 mg/kg (what corresponds to approximately 2000 mg for an average adult) [73].

### 2.4. Loperamide

Is a synthetic opioid antidiarrheal characterized by poor bioavailability and substantial metabolism due to a first pass effect and only a fraction of ingested drug crossing the blood–brain barrier. These traits have contributed to a long-lasting belief that, although a potent opioid (with structural similarity to fentanyl), loperamide is devoid of a central mechanism of action and perfectly safe to use [58,74,75,76]. However, recent reports on its misuse and a sudden increase in number of poisonings recorded since 2014, have led to a re-evaluation of its safety, and prompted a warning from the Food and Drug Administration (FDA) issued in 2016 and regarding arrhythmogenic potential of loperamide when taken in high doses [77,78,79]. The minimal BBB penetration and subsequent insignificant effects on the central nervous system, despite the lipophilic nature of the compound (logP value is estimated to be in the range 4.44–5.5 [80,81]), are a result of P-gp’s activity [82,83]. However, the ingestion of massive amounts, so called megadosing, can lead to drug concentration surpassing P-gp’s processing capacity. Additionally, common agents, such as piperidine (present in black pepper), proton-pump inhibitors (including fellow OTC drug omeprazole), as well as loperamide itself, are P-gp inhibitors with considerable potential to increase the amount of loperamide that is crossing the BBB [78,84]. Additionally, the inhibition of CYP3A4 and CYP2C8 by cimetidine or ranitidine (OTC medications from H_2_-antihistamine class) and the contents of grapefruit juice, have been shown to hinder loperamide’s metabolism, increasing bioavailable drug concentration [75,76,83,84].

Loperamide is an agonist of the µ, δ, and κ opioid receptors. The drug shows strong selectivity towards µ receptors (µ/δ selectivity >300; see Table 1). In therapeutic doses, it almost exclusively binds to a fraction of these receptors located along the gastrointestinal tract. However, when taken in supratherapeutic doses it can manifest its central mechanism of action. Loperamide is also an inhibitor of voltage-dependent P/Q_(1A)_ calcium channels, calcium channels in the intestines, sodium channels in the heart, and calmodulin [75,82,83].

Cardiotoxicity in large doses is visible as a widening of the QRS complex. This is caused by a prolongation in depolarization due to a blockade of sodium channels, and the widening of the QT interval due to prolonged repolarization, which is caused by a blockade of potassium ion channels regulating delayed rectifier currents [74,78,82,85,86,87,88].

Therapeutic dosing typically involves an initial dose of 4 mg, followed by 2 mg after every loose stool (but not exceeding 16 mg a day). A quantity corresponding to 5–10 times the therapeutic dose (20–40 mg) is allegedly enough to alleviate symptoms of opioid withdrawal syndrome, and larger doses of 60–800 mg, or 1600 mg a day, can cause euphoria. However, regardless of consumed amount, loperamide seems to be without an analgesic effect [76,89]. While misuse is more often intended to aid opioid withdrawal rather than for other non-medical purposes, most of the reported poisonings (almost half of them) have been associated with suicide attempts [77,79,90]. As with other opioids, withdrawal syndrome and dependence have also been described with prolonged use of loperamide.

### 2.5. Alternative (Non-Opioid) Antitussives Used to Treat Unproductive Cough

Cough medications stand as one of the most misused drugs, not only within the OTC drugs category, but also among all psychoactive substances [91]. The misuse of antitussives is often associated with the aforementioned opioids, such as codeine or dihydrocodeine, but non-opioid dextromethorphan and zipeprol also gained some recognition as recreational drugs, owing to their hallucinogenic properties when taken in supratherapeutic doses. Several cases of fatal poisonings have since led to a withdrawal of zipeprol. However, dextromethorphan-based cough medicines remain available as one of the most popular antitussive medicines worldwide.

Structurally, dextromethorphan is an isomer of the opioid levorphanol, but due to its marginal affinity to opioid receptors, it is not classified as an opioid. Pharmacologically, dextromethorphan is an agonist of σ receptors (formerly classified as opioid receptors) and, in higher doses, an antagonist of excitatory N-methyl-D-aspartate glutamate receptors (NMDARs). Dextromethorphan is metabolized to dextrophan by the CYP2D6 [92] and, given that this metabolite is credited for the dissociative effects of the drug, in the case of its misuse, dextromethorphan can be considered a prodrug [8].

According to the reports, dextromethorphan is usually misused in quantities of 225–2500 mg [7,93], while a lethal dose is considered to be within the 50–500 mg/kg range (approximately 3500–35,000 mg for an adult) [8]. Such a wide range is mainly an outcome of significant differences to the CYP2D6 activity found across populations, caused by the polymorphism of a related gene [6]. Nevertheless, the aforementioned values result in a drug safety ratio of approximately 15 (other authors state it to be ca. 10 [45]), which is less than a corresponding value for codeine (safety ratio = 20) [45]. This shows that dextromethorphan, although not an opioid itself, is no safer than codeine if not used accordingly.

## 3. Conclusions

The misuse of opioid drugs is an important issue burdening the modern world more than ever before. The scale of this phenomenon, called the 21st century opioid crisis, is clearly illustrated by an ever-increasing involvement of opioids in the total number of all substance overdoses. The number of opioid overdoses has increased nearly 6-fold over the course of the last 20 years, and their contribution to fatal intoxications grew from approximately one half to over two thirds of all cases [94].

While it is generally agreed upon that this problem concerns mainly prescription-only opioids and illicit narcotics (such as novel analogues of fentanyl), the involvement of OTC opioids should not be overlooked as there is sound evidence that such substances are being misused. Relatively easy access to OTC opioids is alarming and perhaps requires additional consideration and debate on the rescheduling of their availability (some countries already undertaken such steps and limited access to codeine or dihydrocodeine [4]). However, as discussed, drugs are among the most popular medicines worldwide, their sudden disappearance from over-the-counter sale could prove problematic for patients. At the moment, codeine and loperamide are both considered essential medicines according to the WHO [95]. However, their misuse liability is an important stimulus for research on new, less-addictive candidates to replace them (both opioid and non-opioid).

## 4. Materials and Methods

### 4.1. Data Source

**Figure 3:**
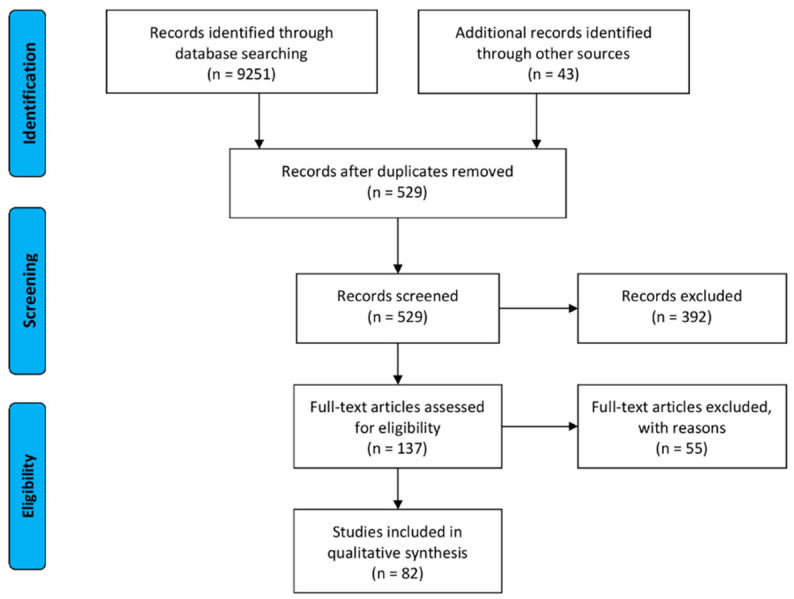
PRISMA Flow Diagram.

Study material was identified and retrieved from the Medline database, accessed via the PubMed website [96], and the full-text databases of the following publishers: Elsevier [97], Springer Nature [98], and Wiley [99]. The search was conducted with phrases created by combining the international non-proprietary names of opioid drugs (codeine, dihydrocodeine, and loperamide), or the word OTC, with terms such as abuse, misuse, addiction, dependence, intoxication, poisoning, and toxicity. An additional search was conducted with the Nicolaus Copernicus University search engine, and references from already included items were screened for previously omitted records. The complete workflow is presented in Figure 3, showing flow diagram prepared according to the PRISMA guidelines [100].

Initially, 9182 items were identified, specifically 5477 for codeine, 393 for dihydrocodeine, 836 for loperamide, and 2476 records discussing OTC drugs in general. Eventually, 137 items were assessed, 43 for codeine, 19 for dihydrocodeine, 20 for loperamide, 41 for opioids, and 14 for OTC drugs.

### 4.2. Inclusion and Exclusion Criteria

As pharmacology was the main focus of this review, no territorial criteria were used. Therefore, records from both counties where disclosed drugs are available without prescription, and those where they are only available with a prescription were included. No limitations regarding publication date were in place; however, when available, recent (less than 5-years-old) and primary literature was prioritized to display the current state of knowledge on the subject. Only papers written in the English and Polish languages were assessed.

### 4.3. Additional Limitations of the Work

The abuse-related substance consumption amounts disclosed in this work are based on user self-reports or toxicological case reports. Thus, presented values only hint at the most popular patterns, and are not establishing the definitive boundaries of this phenomenon. Furthermore, it should be noted that most pharmaceuticals containing the herein discussed substances are formulated using salts of active pharmaceutical ingredients, not their free base or acid forms. As many investigated records failed to mention the exact chemical form, this review further disregarded them, using instead a simplified nomenclature for the sake of unification and clarity.

## Figures and Tables

**Table 1:** Receptor type specific effects of opioid drugs (agonists).

**Receptor Type**	**Main Effects of Receptor Agonism**	**Receptor Agonist**	**Receptor Type Selectivity ^1^**	**Ref.**
**µ** **(mu)**	analgesia bradycardia cough suppression euphoria (rush) miosis (pupil constriction) physical dependence reduced gastrointestinal motility (constipation, cramps) respiratory depression (including decrease in sensitivity of respiratory center for CO_2_) sedation	morphine (reference)	µ/δ: 0.006–0.040 µ/κ: 0.023–0.059	[13,14,15,16]
		codeine (OTC drug)	µ/δ: 0.049–0.051 µ/κ: 0.033–0.044	[13,15]
		dihydrocodeine (OTC drug)	µ/δ: 0.036–0.055 µ/κ: 0.018–0.023	[13,15]
		loperamide (OTC drug)	µ/δ: 0.003	[17]
**δ** **(delta)**	cough suppression (disputed) gastrointestinal dysmotility mood modulation respiratory depression spinal analgesia (pain control)	morphine (reference)	δ/µ: 25.000–159.091	[13,14,15,16]
		SNC80 ^2^	δ/µ: 0.002	[18,19]
		BW373U86 ^3^	δ/µ: 0.120	[20]
**κ** **(kappa)**	cough suppression dysphoria (profound sensation of dissatisfaction and unease) gastrointestinal dysmotility hallucinations peripheral analgesia physical dependence pupil constriction sedation	morphine (reference)	κ/µ: 16.950–42.636	[13,14,15,16]
		butorphanol ^4^	κ/µ: 0.545	[21,22]
		pentazocine ^4^	κ/µ: 0.564–0.772	[14]
		nalorphine ^5^	κ/µ: 0.667–0.895	[14]

^1^ Ratio of Ki-values (lower value = more selective); ^2^ experimental drug (convulsant/antidepressant/anxiolytic); ^3^ experimental drug (convulsant/antidepressant/analgesic); ^4^ therapeutic analgesic; ^5^ opioid overdose antidote.

**Table 2:** Efficacy and toxicity of selected opioids in animal studies.

	**Mouse**					**Rat**				
**Drug**	**LD_50_** **(mg/kg)**		**ED_50_** **(mg/kg)**		**Therapeutic Index**	**LD_50_** **(mg/kg)**		**ED_50_^1^** **(mg/kg)**		**Therapeutic Index**
**Morphine (reference)**	524	[50]	28.8	[51]	18.2–21.2	335	[50]	N/A		N/A
	610	[52]								
**Codeine**	250	[45,53]	43.2	[54]	1.8–5.8	266 427	[45] [53]	69.3	[55]	3.8–6.2
			139.9	[51]						
**Loperamide**	105	[56]	N/A		N/A	185	[56]	0.15	[57]	102–1233
								0.61	[58]	
								1.81	[57,58]	

^1^ Morphine and codeine tested as analgesics, loperamide tested as antidiarrheal.
